# Research Defence Society

**Published:** 1911-02-04

**Authors:** Sydney Holland, F. H. Sandwith, Stephen Paget

**Affiliations:** (President). 21 Ladbroke Square, London, W.; (Chairman of Committee). 21 Ladbroke Square, London, W.; (Hon. Treasurer). 21 Ladbroke Square, London, W.; (Hon. Secretary). 21 Ladbroke Square, London, W.


### Editor's Letter-Box.

### RESEARCH DEFENCE SOCIETY.

*To the Editor of* The Hospital.

Sir,—As it is just three years since the Research Defence Society was formed (Jan. 27, 1908), we hope that you will kindly find room for a short account of its work during the past year. The number of members, which was about 2,800 a year ago, is now 3,770; and the number of associates, which was 130, is now 230. Six new branch societies have been formed, making nineteen in all. Since January last the following have consented to be vice-presidents of the Society: the Bishop of St. Asaph, Miss Balfour, Sir George Beatson, Dr. H. G. Biggs, Sir John Carrington, Lord Hugh Cecil, the Bishop of Chichester, the Earl of Durham, the Bishop of Ely, Sir David Gill, Sir Archibald Hepburn, Lord Kenyon, Professor Leith, the Duke of Norfolk, the Bishop of Nyasaland, Mrs. Henry Sidgwick, the Bishop of Stepney, and Archdeacon Watkins.

Since January last the following pamphlets and leaflets have been published by the Society: (1) Pasteur, Science, and Medicine; (2) The Nation and the Tropics; (3) Plague in India, Past and Present; (4) The Helpfulness of Physiological Experiments to the General Practitioner; (5) Report of Annual General Meeting; (6) Diphtheria and Antitoxin; (7) Inoculations; (8) Experiments during 1909; (9) Charges of Cruelty against the Rockefeller Institute; (10) Fighting the Invisible; (11) What the Doctor Says; (12) The National Welfare; (13) The Brown Dog; (14) A Question of Religion; (15) The Research Defence Society; (16) Sleeping Sickness; (17) Malta Fever; (18) Experiments on Dogs; (19) In Memoriam, Louis Pasteur; (20) Humanity and Science; (21) Friends of Animals.

There has been a very great increase during the past year in the quantity of pamphlets and leaflets distributed, and in the number of addresses and popular lectures, and lantern lectures, given by members of the Society in all parts of the country. Many debates have been attended, also, in London or elsewhere, by the Society's representatives, with excellent results. We are constantly finding that the majority of the audience on these occasions, after hearing both sides, is strongly in favour of the views of this Society.

A large quantity of our publications has been distributed among public libraries and similar institutes; a large quantity, also, is sold in the ordinary way of publication. During the past few months, the Society has been represented by stalls held at the following exhibitions: the Church Congress, the Annual Meeting of the British Medical Association, the Kennel Club Show, and the Birmingham Dog Show.

The Research Defence Society has nothing to do with the actual making of experiments on animals, nor with the administration of the Act; or does it desire to see the abolition of restriction on experiments on animals in this country. It keeps itself to the work for which it was formed three years ago: that is, to make generally known the facts as to experiments on animals in this country and the regulations under which they are conducted, the immense importance of such experiments to the welfare of mankind, and the great saving of human and animal life and health which is already due to them.

We hope that many of your readers will become members or associates of the Society, and will help to extend its educational work.—We remain, Sir, yours faithfully,

Cromer (President).
Sydney Holland (Chairman of Committee).
F. H. Sandwith (Hon. Treasurer).
Stephen Paget (Hon. Secretary).

21 Ladbroke Square, London, W. Jan. 27, 1911.

